# A Review of the Effects of Chronic Arsenic Exposure on Adverse Pregnancy Outcomes

**DOI:** 10.3390/ijerph14060556

**Published:** 2017-05-23

**Authors:** Abul H. Milton, Sumaira Hussain, Shahnaz Akter, Mijanur Rahman, Tafzila A. Mouly, Kane Mitchell

**Affiliations:** 1 Centre for Clinical Epidemiology and Biostatistics (CCEB), School of Medicine and Public Health, Faculty of Health and Medicine, The University of Newcastle, Callaghan 2308, Australia; mdmijanur.rahman@uon.edu.au; 2 Mercy Corps, Pak Palace, Murree Road, Rawal Chowk, Islamabad 44000, Pakistan; sumaira.87@gmail.com; 3 School of Medicine and Public Health, Faculty of Health and Medicine, The University of Newcastle, Callaghan 2308, Australia; shahnaz.akter@uon.edu.au; 4 Centre for Health and Development (CHAD), Dhaka 1219, Bangladesh; tafzila.mouly@yahoo.com.au; 5 Managing Principal, JBS&G, Brisbane 4000, Australia; kmitchell@jbsg.com.au

**Keywords:** arsenic, pregnancy outcomes, chronic exposure, drinking water

## Abstract

Exposure to arsenic has a number of known detrimental health effects but impact on pregnancy outcomes is not as widely recognized. This narrative review examines existing epidemiological evidence investigating the association between arsenic exposure via drinking water and adverse pregnancy outcomes. We reviewed published epidemiological studies from around the world on impact of chronic arsenic exposure on spontaneous abortion, stillbirth, neonatal death, post neonatal death, low birth weight and preterm baby. Plausible mechanisms of arsenic toxicity causing adverse pregnancy outcomes were also determined through literature review. There is convincing evidence to support the association between high inorganic arsenic exposure (>50 ppb) and spontaneous abortion, stillbirth and low birth weight. Limitations of certain studies include study design, small sample size, recall constraints and exposure assessment. There needs to be further research investigating the dose metered impact of arsenic exposure on pregnancy outcomes. Further research on impact of low–moderate arsenic concentration exposure on pregnancy outcomes will allow for appropriate public health policy recommendations.

## 1. Introduction

Over the last few decades, exposure to naturally occurring arsenic in groundwater has emerged as a public health problem [1,2,3]. Globally, more than 120 million people are affected, the majority residing in Bangladesh and India [2,4]. Arsenic is a metalloid element, primarily existing in its inorganic form in water. The toxic impact of arsenic on human health has been documented in numerous studies; to the extent that it has been classified by the International Agency for Research on Carcinogens (IARC) and the National Toxicity Program (NTP) as a known human carcinogen [5,6].

Apart from its cancerous consequences, long-term exposure to arsenic has been associated with developmental effects, cardiovascular disease, neurotoxicity and diabetes [7]. Inorganic arsenic can easily cross human and animal placenta and has been reported to increase the risk of adverse pregnancy outcomes such as spontaneous abortion, stillbirth, impaired fetal growth and infant mortality rate [8]. However, the association between chronic arsenic exposure and adverse pregnancy outcomes is not widely recognized. Convincing evidence is required to stimulate appropriate policy and mitigation measures. This narrative review evaluates the existing epidemiological studies investigating potential linkages and outlines their methodological limitations.

## 2. Methods

A literature search was performed using Ovid MEDLINE, EMBASE, PubMed and Google Scholar databases for identification of relevant studies from 1980 through December 2016. Abstracts of studies that reported exposure to arsenic-contaminated drinking water and adverse pregnancy outcomes were aggregated and the full texts were retrieved. The search terms included: “arsenic”, “spontaneous abortion”, “stillbirth”, “preterm”, “birth weight”, “neonatal death”, “post neonatal death”, “fetal death”, “miscarriage”, “pregnancy outcome”, “pregnancy loss”, and “pregnant”; each term was used in one or more searches. Study selection was limited to those that had direct relevance to human studies of arsenic exposure and pregnancy outcomes. Additional relevant studies were hand-searched and a cross-checked reference list from previous review articles was used for identification of studies not retrieved through the electronic database. We only considered articles published in peer-reviewed journals and in the English language. Each published article selected was critically evaluated and summarized in terms of location, design, pregnancy outcome, sample size, exposure assessment and magnitude of association between exposure to arsenic and pregnancy outcome.

## 3. Mechanism of Arsenic Toxicity for Causing Adverse Pregnancy Outcomes

The mechanisms by which arsenic exposure causes adverse pregnancy outcomes are not completely understood, however, a number of possible mechanisms based on experimental evidence have been suggested. Arsenic occurs either in the elemental form, as part of organic compounds or as a part of inorganic compounds. In waters, inorganic species are dominant [5,9,10]. Inorganic arsenic is eliminated from the body via rapid urinary excretion or a sequential methylation process which predominantly occurs in the liver [4].

Women of childbearing age have a more efficient methylation process compared to other times of their life or in comparison to men [4]. A possible mechanism for this is the estrogen stimulated endogenous production of choline, which is oxidised to betaine and provides a source of methyl groups required for sequential methylation of inorganic arsenic [4,11]. A woman’s methylation efficiency is increased further during pregnancy, in particular the first trimester, via a pathway which appears to be micronutrient independent [12,13]. This suggests that the fetal exposure to arsenic metabolites will vary as the fetus grows (i.e., higher proportion of methylarsonic acid (MMA) during second and third trimesters). As would be expected, variation exists between the extents to which a woman’s methylation efficiency is increased during pregnancy [14].

Inefficient methylation (lower dimethylarsinic acid (DMA) percentages in urine) has been correlated with lower folate levels in blood plasma and elevated homocysteine (Hcy) levels in urine [15]. Elevated Hcy levels are a known risk factor for adverse pregnancy outcomes including neural tube defects, congenital malformations, preeclampsia and placental abruption [4]. This suggests an indirect mechanism by which inorganic arsenic exposure coupled with sub-optimal dietary folate intake elicits a range of adverse pregnancy outcomes.

Although the specific mechanism remains unclear and further research is required, a number of potential mechanisms have been suggested by which arsenic exposure may cause increased spontaneous abortion rates [4,16], namely:

- Arsenites are thiol reactive, which leads to enzyme inhibition and alteration of proteins;
- Inorganic arsenic has been identified as an endocrine disruptor which can interrupt signalling pathways for hormones, including estrogen and progesterone;
- Arsenite elicits cytotoxic effects by altering cell–cell signalling, inducing apoptosis via direct effect on the mitochondrial permeability transition pore and inducing apoptosis via formation of reactive oxygen species.

In vivo human studies have shown that arsenic easily crosses the placenta, particularly during early gestation, although there is conflicting evidence available regarding the form of arsenic which reaches the placenta [4]. The transplacental nature of arsenic has been suggested as a mechanism for adverse pregnancy outcomes including spontaneous abortion and low birth weight. Diminished nutrient supply as a consequence of arsenic driven vasoconstriction has been suggested as a mechanism for relatively low birth weight [17]. Relatively inefficient methylation by the mother has also been suggested as a potential cause of relatively low birth weight [12]. Arsenite and MMA^3+^ have been identified as immunosuppressors, possibly due to the inhibition of heme synthesis. The immunosuppressive effects of arsenite and MMA^3+^ may represent a mechanism for increased infant mortality rates [4].

## 4. Epidemiological Studies

Various epidemiological studies were identified that have investigated the association between arsenic exposure via drinking water and adverse pregnancy outcomes. Table 1 summarizes these studies with the following specific pregnancy outcomes: spontaneous abortion, stillbirth, preterm birth, neonatal death, and post neonatal death. Similarly, Table 2 summarises studies that investigated the association of arsenic exposure with low birth weight. Comparisons across different studies is challenging as data variables are not identical in definition, including variability in definitions of spontaneous abortion (loss prior to 20–28 weeks of gestation), stillbirth (loss following 20–28 weeks of gestation), along with variability in denominators, such as reproductive age, pregnant women, live birth, etc. Nevertheless, existing evidence consistently supports of the association between higher arsenic exposure and adverse pregnancy outcomes. Abbreviated terms used include confidence interval (CI), odds ratio (OR), relative risk (RR), probability (p), parts per billion (ppb) and interquartile range (IQR).

### 4.1. Spontaneous Abortion and Stillbirth

A number of epidemiological studies from Bangladesh, India, Sweden, Hungary, Mongolia, and the United States of America (USA) have reported an increased risk of spontaneous abortion (loss of a clinically recognised pregnancy prior to 20 weeks completed gestation) and stillbirth (loss of a clinically recognised pregnancy following 20 weeks completed gestation) in association with chronic arsenic exposure. The literature includes 12 studies assessing the association of arsenic exposure with spontaneous abortion, including: seven cross-sectional studies, two case-control studies, two ecologic studies and two retrospective cohort studies. Of these, eight support an increasing risk with greater exposure, three observed no association and one did not report on any association. In addition, there are 12 studies assessing the association of arsenic exposure with stillbirth, including: seven cross-sectional studies, three ecologic studies and two retrospective cohort studies. Of these, nine support an increasing risk with greater exposure, one observed no association, one observed a decreasing risk with greater exposure and one did not report any association.

In southeast Hungary, an ecological study of approximately 8000 pregnancies and 5000 events was conducted among women residing in an arsenic contaminated region between 1980 and 1987 [18]. Women exposed to arsenic in drinking water at levels typically >100 ppb were compared to a group of women with relatively low arsenic exposure. Women exposed to higher levels of arsenic were more likely to have reported a higher rate of spontaneous abortion (prevalence OR: 1.36, *p* < 0.01) and stillbirth (prevalence OR: 2.79, *p* < 0.05).

Similarly, a large ecological study in Chile reported on several thousand pregnancy outcomes occurring in Antofagasta, a city with an arsenic concentration of 50–860 ppb in drinking water and Valparaiso, a city with similar demographics but with an arsenic concentration of <5 ppb in drinking water. The study found a 70% increase in the risk for stillbirth (RR: 1.70, 95% CI: 1.50–1.90) among the residents of Antofagasta, who were exposed to higher concentrations of arsenic [19].

A cross-sectional study conducted in Mongolia found a positive association between arsenic exposure and spontaneous abortion when comparing 224 women residing in villages contaminated with arsenic >50 ppb in drinking water to 99 women residing in villages with low arsenic concentration of <50 ppb in drinking water. Among the group with higher levels of arsenic exposure, an increased OR of 2.70 (95% CI: 0.80–8.40) was observed for spontaneous abortion; however, this difference was not statistically significant [20].

Whereas the previous studies drew conclusions based on comparison data from residents exposed to relatively high levels of arsenic in drinking water, a hospital based case-control study conducted in Massachusetts, USA from 1976 to 1978, attempted to assess the association between spontaneous abortion and low to moderate levels of arsenic exposure (<10 ppb) through municipal drinking water supply [21]. In this study, an increased odds ratio for spontaneous abortion was observed among women exposed to an arsenic level of 0.80–1.30 ppb (OR: 1.20, 95% CI: 1.00–1.60), or 1.40–1.90 ppb (OR: 1.70, 95% CI: 0.70–4.20), compared to women exposed to arsenic <0.80 ppb. However, these associations were reduced and statistically insignificant after adjusting for 19 covariates (OR: 1.10, 95% CI: 0.60–1.80 and OR: 1.50, 95% CI: 0.40–4.70, respectively). For adjusted analysis, individual arsenic exposure during pregnancy was assessed based on publicly available utility records consisting of the water analysis results as part of the federally mandated routine drinking water monitoring activities.

A number of studies investigating the association between chronic arsenic exposure and spontaneous abortion have been reported from Bangladesh and India, where more than 122 million people depend on hand wells frequently contaminated with arsenic for water consumption [27]. Excess spontaneous abortion and stillbirth was first reported from Bangladesh in a cross-sectional study, where pregnancy outcomes of 96 women using drinking water with arsenic concentration >50 ppb were compared to 96 women using drinking water with arsenic concentration ≤20 ppb. Those women exposed to higher arsenic concentrations had a 2.9 and 2.2 times (*p* = 0.008, *p* = 0.046) increased risk of spontaneous abortion and stillbirth when compared to those exposed to lower arsenic concentrations [22].

A small cross sectional study of 22 arsenic exposed and 18 unexposed women in the Uttar Pradesh, India, revealed an increased incidence of reported spontaneous abortion and stillbirths (200 spontaneous abortion and 250 stillbirth/1000 live births for 201–500 ppb, 268 spontaneous abortion and 171 stillbirths/1000 live births for 501–1200 ppb) compared to an urban and sub-urban population exposed to very low arsenic in drinking water (170 spontaneous abortion and 51 stillbirths/1000 live births) [23].

A large cross-sectional study of 533 women aged 15–49 years in rural Bangladesh also reported an increased risk of spontaneous abortion and stillbirth for arsenic exposure over 50 ppb in drinking water. In this study, a 2.50-fold increase (95% CI: 1.50–4.30) in self-reported spontaneous abortion and a 2.50-fold increase (95% CI: 1.30–4.90) in stillbirth was observed among the group of women who had been exposed to higher levels of arsenic (>50 ppb) when compared to lower concentrations of arsenic (<50 ppb) in drinking water. Risks were higher with longer duration of arsenic exposure for both spontaneous abortion and stillbirth [24].

On the other hand, another cross-sectional study of three sub-districts of rural Bangladesh did not observe any association between arsenic exposure and stillbirth (OR: 0.996, 95% CI: 0.996–1.002) among 2006 women. Study participants were contacted and interviewed in 2003 regarding their pregnancies that had been completed in 2002. Individual arsenic exposure was determined by collection of water from the main drinking sources during the time of interview [17].

An increased risk of stillbirth in relation to exposure to arsenic in drinking water was reported from a large ecological study conducted in 200 villages in Bangladesh between 2001 and 2003. These villages were administered from 16 geographical centres, with 30,984 pregnancies and outcomes recorded along with information on 26 variables related to socio-economic and health status of the participants. Average arsenic concentration for each centre was obtained from the National Hydrochemical Survey. The study reported an overall stillbirth of 3.40% which increased with estimated arsenic concentration (2.96% at <10 ppb, 3.79% at ≤50 ppb, 4.43% at ≥50 ppb). In an adjusted analysis for 17 socioeconomic and health related variables, the OR for stillbirth remained high, with OR: 1.23 (95% CI: 0.87–1.74) at ≤50 ppb and OR: 1.8 (95% CI: 1.14–2.86) at ≥50 ppb compared to arsenic exposure <10 ppb [25].

A retrospective cohort study conducted in Matlab, Bangladesh, on pregnancy outcomes of 29,134 women between 1991 and 2000, reported an increased risk of fetal loss inclusive of spontaneous abortion and stillbirth. Exposure to arsenic in drinking water >50 ppb during pregnancy increased the rate of fetal loss by a factor of 1.10 (95% CI: 1.04–1.25) compared to women exposed to water with arsenic concentration of <50 ppb. Pregnancy outcome data was obtained from the monthly health and demographic surveillance system in Matlab. Data from a separate survey conducted in 2002–2003 analyzing arsenic concentrations in tube-well water in Matlab was used to determine arsenic exposure data after correlation with drinking water history of participants during pregnancy [26].

A small cross-sectional study consisting of 18 female participants in West Bengal, India reported an increased proportion of spontaneous abortion among women exposed to drinking water with arsenic concentration of 401–1474 ppb (182 spontaneous abortions/1000 pregnancies) compared to women exposed to 200–400 ppb (95 spontaneous abortions/1000 pregnancies). An inverse relationship was observed when investigating for stillbirths, as those women who were exposed to lower concentrations of arsenic in drinking water had a higher proportion of stillbirths (158 stillbirth/1000 live births) compared to women exposed to high arsenic concentrations (55 stillbirths/1000 live births). This observed association may be due to the small sample size or the possibility that higher exposure to arsenic would expedite the toxic impact on pregnancy, resulting in spontaneous abortion whereas moderate exposure to arsenic would take longer to have an effect, resulting in stillbirth [27].

A larger cross-sectional study in West Bengal, India, with 202 women participants totaling 644 pregnancies, did not find any association between drinking water arsenic exposure and spontaneous abortion (OR: 1.01, 95% CI: 0.38–2.70). However, a positive association was reported between drinking water arsenic exposure and stillbirth after adjusting for potential confounders. The risk of stillbirth was 6.07 times higher (95% CI: 1.54–24.00) among women exposed to arsenic concentration ≥200 ppb compared to women exposed to arsenic concentration <50 ppb. Arsenic exposure during pregnancy for each of the participants was determined through a comprehensive strategy utilizing interview and laboratory data [28].

Another small cross-sectional study in villages of West Bengal, India, found statistically higher (*p* < 0.05) rates of spontaneous abortion and stillbirth among women exposed to arsenic in drinking water between 10 and 600 ppb compared to women exposed to <10 ppb. This study included 240 female participants from four villages with high arsenic concentrations in drinking water and 60 female participants from a village with a low arsenic exposure [29].

A hospital-based case-control study did not observe a statistically significant association between low–moderate level exposure to drinking water arsenic (0.00 to 175.10 μg/L, with median 0.40 μg/L and 90th percentile 9.40 μg/L) and spontaneous pregnancy loss of <20 weeks completed gestation [30]. On the other hand, a small study investigated pregnancy outcomes of four women suffering from chronic arsenic toxicity in Patna District of India, where arsenic concentration was more than 10 mg/L in 61% hand tube-wells and above 50 mg/L in 44%. Among a total of 19 pregnancies, the outcomes are as follows: four stillbirth, five spontaneous abortion, four neonatal death and two preterm birth [31].

Collectively, the majority of studies support the positive association between arsenic exposure and spontaneous abortion. The studies range in sample size from 18 to hundreds of thousands. The most frequently employed study design was cross-sectional. Aside from three studies, all had adjusted associations for confounders. Of the three studies that reported no association, two measured the association between low–moderate arsenic exposure (0–2 ppb; 0.4 ppb) and spontaneous abortion. The remainder of the studies reported on associations based on higher arsenic exposure (>10 ppb). In addition, of the 12 studies assessing the association of arsenic exposure with stillbirth, nine supported a positive association.

### 4.2. Neonatal and Post Neonatal Death

Only a few studies have been conducted exploring the association between chronic arsenic exposure and neonatal death (mortality within the first 28 days following delivery) and post neonatal death (mortality between 28 days and 364 days following delivery). The literature includes one cross sectional study and one ecologic study. Although one study suggests a positive and statistically significant association between arsenic exposure and neonatal/post-neonatal death, this area requires further evidence to support this association. Milton et al., in their study on chronic arsenic exposure and adverse pregnancy outcomes in Bangladesh, observed a positive but statistically insignificant association (OR: 1.80, 95% CI: 0.90–3.60) between chronic arsenic exposure and neonatal death [24]. Whereas a study from Chile compared the trends in infant mortality between 1950 and 1996 among two geographical areas: Antofagasta, with a well-documented history of arsenic exposure from naturally contaminated water and Valparaiso, with low arsenic contamination. The study reported an increased and statistically significant association between arsenic exposure and late fetal mortality (RR: 1.70, 95% CI: 1.50–1.90), neonatal mortality (RR: 1.53, 95% CI: 1.40–1.70) and post neonatal mortality (RR: 1.26, 95% CI: 1.20–1.30) rates for Antofagasta compared to Valparaiso, particularly during the time period when Antofagasta had high arsenic concentration in drinking water [19].

### 4.3. Low Birth Weight and Preterm Birth

There have been several studies reporting on the association between arsenic exposure and low birth weight (birth weight of less than 2500 g) and preterm birth (live delivery prior to 37 weeks completed gestation). The literature includes 11 studies assessing the association of arsenic exposure with low birth weight, including: seven prospective cohort studies, one retrospective cohort study, one cross sectional study and one longitudinal study. Of these, six support an increasing risk with greater exposure while four did not observe a statistically significant association. In addition, the literature includes three studies assessing the association of arsenic exposure with preterm birth, including: one cross-sectional study and two retrospective cohort studies. Of these, one supports an increasing risk with greater exposure while two did not observe a statistically significant association.

A prospective cohort study conducted in two Chilean cities with contrasting drinking water arsenic levels, Antofagasta (40 ppb) and Valparaiso (<1 ppb), found that moderate arsenic exposure from drinking water (<50 ppb) during pregnancy was associated with low birth weight [36]. A study from Dalian, China, suggests that maternal exposure to arsenic is associated with impaired fetal growth. Maternal blood arsenic concentration was inversely associated with birth weight, height and chest circumference in newborns. Furthermore, fetal cord blood arsenic concentration was negatively associated with head circumference. To validate this observed association, other factors could have been measured including, maternal urinary arsenic concentration and potential sources of arsenic exposure [37]. A similar study in Shanghai, China, investigating the effects of low maternal arsenic exposure during pregnancy on fetal growth suggests that even low arsenic exposure negatively influences a newborn’s birth weight. Interestingly, this effect was observed only in male babies, suggesting a sex differential in susceptibility to arsenic during the early stages of development [38].

A study in the northeastern Taiwan compared the risk of preterm delivery and low birth weight between an area with historic high arsenic concentration in well water and an area with no historic evidence of arsenic contamination in drinking water. Although the study indicated an increased risk of preterm delivery among the arsenic exposed population compared to the unexposed population, the observed association was not statistically significant (OR: 1.10; 95% CI: 0.91–1.33). The study also reported lower newborn birth weight by 29.05 g (95% CI: 13.55–44.55) among the arsenic-exposed pregnant women compared to the unexposed participants [39]. A study in Bangladesh suggests that maternal arsenic exposure during early pregnancy is inversely associated with newborns’ birth weight. The study measured arsenic in pregnant mothers’ hair, toenail and drinking water sources at multiple times during pregnancy and from their newborns after birth. The study also concluded that maternal hair provided the best integrated measure of arsenic exposure [40].

More recently, a prospectively enrolled cohort study in Bangladesh studied 1140 pregnant women from 2008 to 2011. Arsenic exposure was measured through maternal drinking water source at the time of enrollment (gestational age < 16 weeks) and through maternal toenail clippings, collected during pregnancy to assess the cumulative exposure across the perinatal period (*n* = 624). A 19.17 g (95% CI: −24.64, −13.69) birth weight decrease with every unit increase in natural log water arsenic was observed. The result was similar for maternal toenail clippings with 15.72 g (95% CI: −24.52, −6.91) weight decreased for every unit increase in natural log toenail arsenic. Overall, this study found newborn birth weight decreased by 16–19 g for every unit increase in natural log-transformed arsenic in both drinking water and maternal toenail [41]. Comparably, a study conducted near a mining-related Superfund site in Northeast Oklahoma among 622 mother–infant pairs found that an interquartile range increase in maternal blood arsenic was consistently negatively associated with −77.50 g (95% CI: −127.80, −27.30) birth weight [42].

While investigating newborn birth weight among mothers who smoked and were exposed to arsenic, results specified that higher level arsenic exposure (10 g/L) in smokers was associated with a −2.45 decrease in birth weight Z-score (*p*: 0.02) but not in non-smokers or smokers with low arsenic exposure [32]. In contrast, Gilbert-Diamond and colleagues [33] found no association between birthweight and maternal urinary arsenic level in 706 mother–infant pairs of a cohort from New Hampshire. However, in sub-group analysis, there was a statistically significant negative association of −90.7 g (95% CI: −161, −20.5) between arsenic exposure and birth weight of female infants born of overweight/obese mothers. Moreover, Bloom et al. [34] and Röllin et al. [35] did not observe a statistically significant association between low birth weight and arsenic exposure in a longitudinal study of 501 couples from central Michigan and 650 women in South Africa respectively. However, in a subgroup analysis conducted by Bloom et al., there was a statistically significant positive association between paternal arsenic exposure and birth weight.

Collectively, of the 11 studies assessing the association of arsenic exposure with low birth weight, six support a positive association. The four studies that reported a statistically insignificant association had two study designs: longitudinal and prospective cohort. In addition, the exposure measure and mean exposure varied, whereas the sample size was consistently more than 200 participants. There was a noteworthy element in the subgroup analysis of each of these studies, elucidating future research opportunities, especially in regards to investigating whether arsenic has any gender affinity.

## 5. Limitations to Existing Evidence

There are limitations affecting the strength of the results derived from several of the reviewed epidemiological studies investigating the association between chronic arsenic exposure and pregnancy outcomes. These limitations include: study design, small sample size, recall constraints and exposure assessment. The vast majority of the published studies reviewed here used a cross-sectional study design, followed by ecologic study design.

Cross-sectional study designs are effective in determining the justification or refutability of a hypothesis. However, what is lacking is their ability to ascertain a temporal sequence (exposure prior to outcome) which prevents the establishment of causality. In addition, in most of the cross-sectional studies, interviews were conducted with participants to gather data retrospectively regarding drinking water sources and pregnancy events (spontaneous abortion, stillbirth). This introduces both recall bias and misclassification error. Furthermore, several cross-sectional studies were conducted using small sample sizes with limited pregnancy events occurring, thereby limiting the precision of rates determined.

Conversely, the ecologic studies reviewed catered to large populations, measuring groundwater arsenic concentration according to geographic regions or during an extended interval of time at one location. Although this provided relatively precise estimated rates for pregnancy events, the inability to distinguish data between individuals gave rise to numerous biases, most notably ecologic fallacy.

Exposure assessment has always been a challenge in environmental epidemiology. In most of the studies, arsenic exposure assessment was ecologic in nature, where the single nearest/most consumed drinking water source of a participant was measured for arsenic contamination. In some instances, these measurements occurred years after the pregnancy. There was some degree of misclassification error present due to differences in arsenic concentration of drinking water sources with varying geographical locations as well as gaps in time after which measurement occurred.

Some of the studies derived their associations by measuring arsenic exposure through urinary arsenic concentrations, which only assesses recent exposure, whereas other studies measured only blood arsenic concentrations in which half-life of inorganic arsenic is 3–4 h [9]. These associations could have been further validated if more than one means of measuring arsenic exposure was employed. Furthermore, the chronicity of arsenic exposure was not determined in all studies, which is an important factor to consider while deriving conclusions on pregnancy outcomes.

## 6. Conclusions

Despite the limitations prevalent among the reviewed studies, there is consistent and convincing evidence present to acknowledge a positive association between exposure to high concentration of inorganic arsenic exposure (>50 ppb) in drinking water and spontaneous abortion, stillbirth and low birth weight. There are too few studies assessing the association of arsenic exposure with neonatal death and preterm birth; this knowledge gap needs to be addressed through further research. The majority of the existing literature derived association assumptions based on populations exposed to high concentrations of arsenic. To date, there remains a paucity of research investigating the impact of low–moderate arsenic exposure on pregnancy outcomes. This is critical as the majority of the world population falls under this category and data regarding pregnancy outcomes from high arsenic exposure groups cannot accurately be extrapolated to low–moderate arsenic exposure groups. Therefore, large prospective cohort studies with comprehensive arsenic exposure ranges are recommended to provide valid data that can be used to determine mitigation strategies. This accomplishment will serve as a platform for the protection of countless pregnant women exposed to arsenic-contaminated drinking water.

## Figures and Tables

**Table 1:** Summary of epidemiologic studies on drinking water arsenic exposure and adverse pregnancy outcome (spontaneous abortion, stillbirth, preterm birth, neonatal mortality, and post-neonatal mortality).

Authors	Location	Design	Pregnancy Outcome	Sample Size	Exposed Group (ppb)	Reference Group (ppb)	Adjusted Association	Rate Ratio	*p*-Value/CI
Börzsönyi et al., 1992 [18]	Hungary	Ecologic	Spontaneous abortion, Stillbirth	7847	High	Low	No	1.36 2.79	0.0071 0.0283
Hopenhayn-Rich et al., 2000 [19]	Chile	Ecologic	Stillbirth, Neonatal mortality, Post neonatal mortality	Hundreds of thousands	50–860 over time	<5	Yes	1.7 1.53 1.26	1.5–1.9 1.4–1.7 1.2–1.3
Guo et al., 2003 [20]	Mongolia	Cross-sectional	Spontaneous abortion	323	>50	<50	Yes	2.7	0.8–8.4
Aschengrau et al., 1989 [21]	USA	Case-control	Spontaneous abortion	1677	0.8–1.3 1.4–1.9	<0.8 <0.8	Yes	1.1 1.5	0.6–1.8 0.4–4.7
Ahmad et al., 2001 [22]	Bangladesh	Cross-sectional	Spontaneous abortion, Stillbirth Preterm birth	192	>50	≤20	Yes	2.9 2.2 2.5	0.008 0.046 0.018
Ahamed et al., 2006 [23]	India	Cross-sectional	Spontaneous abortion, Stillbirth	40	201–500	~0	Yes	1.18 4.90	- -
Milton et al., 2005 [24]	Bangladesh	Cross-sectional	Spontaneous abortion, Stillbirth, Neonatal mortality	533	≥50	<50	Yes	2.5 2.5 1.8	1.5–4.3 1.3–4.9 0.9–3.6
Kwok et al., 2006 [17]	Bangladesh	Cross-sectional	Stillbirth	2006	>300	<10	Yes	0.999	0.996–1.002
Cherry et al., 2008 [25]	Bangladesh	Ecologic	Stillbirth	31,000	≤50 ≥50	<10	Yes	1.23 1.8	0.87–1.74 1.14-2.86
Rahman et al., 2007 [26]	Bangladesh	Retrospective Cohort	Any loss	29,134	>50	<50	Yes	1.14	1.04–1.25
Chakraborti et al., 2004 [27]	India	Cross-sectional	Spontaneous abortion, Stillbirth	18	401–1474	200–400	No	1.92 0.35	<0.05 <0.05
von Ehrenstein et al., 2006 [28]	India	Cross-sectional	Spontaneous abortion, Stillbirth	644	≥200	<50	Yes	1.01 6.07	0.38–2.70 1.54–24.0
Sen and Chaudhuri, 2008 [29]	India	Cross-sectional	Spontaneous abortion, Stillbirth	300	10–600	<10	Yes	1.75 2.38	<0.05 <0.05
Bloom et al., 2014 [30]	Romania	Case-control	Spontaneous abortion	300	0–175	0–175	-	0.98	0.96–1.01
Chakraborti et al., 2016 [31]	India	Retrospective cohort	Spontaneous abortion, Stillbirth, Neonatal mortality, Preterm birth	19	10–50	<3	No	Not reported	Not reported

**Table 2:** Epidemiologic studies of arsenic exposure and low birth weight.

Authors	Location	Design	Pregnancy Outcome	Sample Size	Exposure Measure	Exposed Group/Mean Exposure	Reference Group	Adjusted Association	Birth Weight/Unit Increase in Exposure Measure	*p*-Value/CI
Bloom et al., 2016 [32]	Romania	Prospective cohort	Birth weight	122	Drinking water	10 μg/L	-	-	−2.45 lower birth weight Z-score	0.02
Gilbert-Diamond et al., 2016 [33]	USA	Prospective cohort	Birth weight (male) Birth weight (female)	706	Maternal urine	3.4 (1.7–6.0) μg/L	-	Yes	-	-
Bloom et al., 2015 [34]	USA	Longitudinal	Birth weight	215	Maternal urine	17.13 (−11.63–45.89) μg/L	-	yes	−23.75 g	−199.00–151.50
Röllin et al., 2017 [35]	South Africa	Prospective cohort	Birth weight	650	Maternal blood	0.62 μg/L (0.58–0.66)	-	yes	−0.071 g	−0.386–0.244
Hopenhayn et al., 2003 [36]	Chile	Prospective cohort	Birth weight	844	Drinking water	40	<1	Yes	−57 g	−123–9 g
Guan et al., 2012 [37]	China	Cross-sectional	Birth weight	125	Arsenic in maternal blood	Arsenic affected area (590 ppb)	arsenic free area	Yes	-	-
Xu et al., 2011 [38]	China	Cross-sectional	Birth weight (male) Birth weight (female)	142	Maternal whole blood (μg/L)	4.13 ± 3.21	-	Yes	−0.288 −0.001	<0.05 >0.05
Yang et al., 2003 [39]	Taiwan	Retrospective cohort	Preterm birth Birth weight	18,259	-	Exposed area (0–3590 μg/L)	non-exposed area	Yes	1.1 −29.05 g	0.91–1.33 −44.55–13.55
Huyck et al., 2007 [40]	Bangladesh	Prospective cohort	Birth weight	49	Maternal hair	≥2.70 μg/g	<0.28 μg/g	Yes	−193.5 g	−283.5–103.5
Kile et al., 2016 [41]	Bangladesh	Prospective cohort	Birth weight	1140	Drinking water	2.3 (IQR: 0.9, 36 μg/L)	-	Yes	−19.17g	−24.64–13.69
Henn et al., 2016 [42]	USA	Prospective cohort	Birth weight	622	Maternal whole blood	1.4 (1.0–2.3) μg/L	-	-	−77.50 g/IQR increase	−127.8–−27.3
